# Application of high frequency color Doppler ultrasound in the monitoring of rheumatoid arthritis treatment

**DOI:** 10.3892/etm.2014.2001

**Published:** 2014-10-01

**Authors:** GUIMIN ZHENG, LEI WANG, XIUCHUAN JIA, FANG LI, YONGLONG YAN, ZHIBO YU, LI LI, QUN WEI, FENGXIAO ZHANG

**Affiliations:** 1 Department of Rheumatology, Hebei Province General Hospital, Shijiazhuang, Hebei 050051, P.R. China; 2 Department of Medicine Imaging, Hebei Province General Hospital, Shijiazhuang, Hebei 050051, P.R. China; 3 Department of Ultrasound Imaging, Hebei Province General Hospital, Shijiazhuang, Hebei 050051, P.R. China; 4 Department of Nosocomial Infection Control, Hebei Province General Hospital, Shijiazhuang, Hebei 050051, P.R. China

**Keywords:** rheumatoid arthritis, score of disease activity, metacarpophalangeal joint, wrist joint, high frequency ultrasound, Doppler ultrasound

## Abstract

The aim of the present study was to explore the use of high frequency color Doppler ultrasound to measure synovial thickness and blood flow to assess the therapeutic value of the recombinant human tumor necrosis factor (TNF) II receptor antibody fusion protein in rheumatoid arthritis (RA) treatment. A total of 36 clinically-diagnosed patients with RA were treated with methotrexate tablets or the recombinant TNF-receptor antibody fusion protein for 24 weeks. Joint synovial thickness and synovial blood flow integrity were monitored by high frequency color Doppler in the second metacarpophalangeal joint in one hand. The correlation of the erythrocyte sedimentation rate, C-reactive protein (CRP) and 28-joint disease activity score (DAS28) with the ultrasound parameters were analyzed. Metacarpophalangeal second joint 2 (MCP2) synovial thickness, wrist joint synovial thickness and MCP2 synovial blood flow, prior and subsequent to the treatment, have a high correlation with DAS28 (P<0.05), and the MCP2 synovial blood flow integral has a strong correlation with CRP. Evaluating the wrist joint synovial thickness and synovial integrity of the second metacarpophalangeal joint using high frequency ultrasound detection can effectively evaluate the disease status in patients with RA. This procedure is potentially valuable as a means of evaluating the curative effects of RA treatments.

## Introduction

Rheumatoid arthritis (RA) is a form of autoimmune arthritis that can cause disability and pathological manifestations of synovitis that most often involves the finger joints in the hands and the wrist joint. In clinical practice, assessment of RA progression often requires clinical evaluation, inflammatory index measurement and imaging of the rheumatoid. These parameters contribute to the choice of treatment and are used in treatment monitoring and prognosis (1). Currently, the European League Against Rheumatism (EULAR) recommends that RA is evaluated using a 28-joint disease activity score (DAS28) evaluation of 28 joints and this has become the most accepted evaluation method by the majority of clinicians (2). However, this method can only evaluate the articular inflammatory degree indirectly. Since high frequency color Doppler ultrasound can measure synovial thickening, monitor joint effusion and simultaneously observe changes in the blood flow in the synovium, it is often used in the early diagnosis of RA (3). Additionally, high frequency ultrasound can also monitor disease status, evaluate efficacy of treatments and predict disease recurrence (4). To accurately evaluate the RA disease status, progression and prognosis, more precise quantitative indexes are required. Currently, numerous investigators have proposed their own set of quantitative evaluation criteria, which mainly include the qualitative and quantitative evaluation of synovitis and bone erosion. However, these criteria are not uniform, require the simultaneous detection of a number of joints, are cumbersome and cannot be easily applied in a clinical practice. The present study developed a relatively simple and quick ultrasound evaluation method that focused on the second metacarpophalangeal joint and wrist of the disease-involved side. Using this evaluation method, the ultrasonography data was correlated with other clinical indexes (such as DAS28) prior and subsequent to treatment with a standard anti-inflammatory medication [tumor necrosis factor (TNF)-receptor fusion protein].

## Materials and methods

### Subject

Between September 2011 and January 2013, 36 patients that were admitted to the Hebei People’s Hospital (Shijiazhuang, China) and diagnosed with RA in accordance with the RA classification standard of the American Rheumatism Association and the European League Against Rheumatism (2010) (5), were selected for the study. These patients presented with inflammation of at least one side of the second metacarpophalangeal joint, and had wrist joint swelling and pain. A total of 32 patients were female and four patients were male. All the patients were between 19–65 years old, and averaged 41.5 years old. The patients had presented with disease prior to the study for six months to 21 years, with a mean of 8.2 years. The study was conducted in accordance with the declaration of Helsinki and with approval from the Ethics Committee of Hebei Province Hospitals. Written informed consent was obtained from all the participants.

### Preparation of research objectives

All the patients were administered methotrexate tablets (10–15 mg/week) or combined injection of recombinant human TNF-receptor antibody fusion protein (etanercept; CP Guojian Pharmaceutical Co. Ltd. Shanghai, China) for injection at 25 mg/2 weeks of treatment, combined with non-steroid anti-inflammatory analgesic drug treatment, allowing for systemic or intra articular corticosteroid therapy. The duration of treatment was 24 weeks.

### Detection of clinical indexes

The clinicians in the Department of Rheumatism recorded the following information for each patient: The swollen joint count of 28 joints (SJC28, including the bilateral shoulder, elbow, wrist, metacarpophalangeal, and proximal interphalangeal joints), the tender joint count (TJC28), joint pain using the visual analogue scale (VAS) and the erythrocyte sedimentation rate (ESR). The ESR was measured using the Westergren test, in which healthy males have an upper limit of 15 mm/h, and healthy females have an upper limit of 20 mm/h. The following formula was used to calculate the DAS28 score (2), in which T28 is the number of painful swollen joints from 28 joints and SW28 is the number of swollen joints from 28 joints:

DAS28=0.56×SQRT (T28)+0.28×SQRT (SW28)+0.70×ln (ESR)+0.014×VAS

The nephelometry Siemens BNII special protein analyzer reagents were provided by Behring Diagnostics Products (Shanghai Co., Ltd., Shanghai, China) and were used to measure the C-reactive protein (CRP) in the peripheral blood. Healthy individuals have a CRP level with an upper limit of 10 mg/l.

### Ultrasonography of specific joints

The hands of the patient were stretched flat on the examining table. The second metacarpophalangeal finger joint and the wrist joint of the hand with the more pronounced swelling were fixed and imaged using a GE Voluson Expert 730 type dynamic three dimensional color ultrasound diagnostic apparatus (GE Healthcare, Princeton, NJ, USA), with a 5–12 MHz high frequency linear array probe and a 5–12 MHz dynamic three-dimensional probe. Under specific instrument settings, two-dimensional ultrasonography was performed by transversely and longitudinally scanning the dorsal and palmar metacarpophalangeal joint (metacarpophalangeal second joint 2; MCP2), and wrist joint. The tendon, synovium, articular cavity, cortical bone and blood flow was observed.

### Specific indexes of ultrasonography

**Figure 1:**
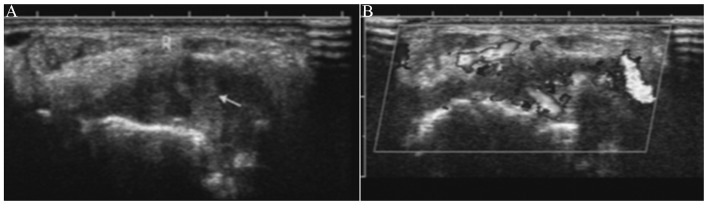
(A) Synovial thickness of the second metacarpophalangeal joint (arrow represents hyperplasia of the synovium). (B) Level 3 synovitis of the second metacarpophalangeal joint in color Doppler.

The thickest section of the synovium was measured three times, taking the mean value (Fig. 1A shows the synovial thickness of the second metacarpophalangeal joint); simultaneously, the tendon sheath was thickened and indicated effusion.

Under the Power Doppler conditions, the thickest synovial section was selected to observe and measure synovial blood flow (Fig. 1B shows the level 3 synovitis of the second metacarpophalangeal joint in color Doppler). Semi-quantitative evaluation of synovium blood flow was divided into four levels: Level 0 had no blood flow signal within the synovial membrane; level 1 had one to two punctiform signals; level 2 had three to four strip signals of blood flow and a distribution of not more than one half of the synovial surface; and level 3 has a dendritic, and a network blood flow signal and distribution over one-half of the synovial surface (6). Semi-quantitative ultrasound examinations were performed on all the patients by the same experienced ultrasound doctors who were blinded, as they had no prior knowledge of the clinical history of the patients.

### Statistical analysis

SPSS 19 (SPSS, Inc., Chicago, IL, USA) was used for statistical analysis. Data are presented as the mean ± standard deviation. Comparisons between the two groups of normal distribution were performed using paired samples t-test, and abnormal distribution data were compared using a two-sample rank sum test. Ultrasonic synovitis thickness measurements, blood flow scores, DAS28, CRP and ESR were compared by correlation analysis, where data with a normal distribution was compared using the Pearson correlation test, and abnormally distributed data were compared using the Spearman correlation test, with P<0.05 considered to indicate a statistically significant difference.

## Results

### Patient characteristics

A total of 29 patients completed the 24 weeks of observation and were enrolled into the study. Of these, 25 patients were female and four were male, aged 19–65 years, with a mean age of 40.5 years. The course of disease was six months to 21 years, with a mean of 7.8 years. In total, 20 patients were treated by injection of recombinant human TNF-receptor antibody fusion protein 25 mg/2 weeks combined with methotrexate tablets (10–15 mg/week). Nine patients were treated with a single dose of methotrexate (10–15 mg) and seven patients withdrew from the study due to an adverse drug reaction.

### Clinical, laboratory and ultrasonic course

All the laboratory data, clinical data and ultrasound results obtained from the 29 patients prior and subsequent to the treatment are listed in Table I. DAS28, CRP and ESR increased prior to treatment, indicating that the RA of these patients was in the active phase (with mean values of 6.10±0.90, 16.20±25.77 mg/l and 34.00±30.00 mm/h, respectively). After treatment for 24 weeks, the overall condition of the 29 patients improved and a number of the disease parameters improved significantly.

MCP2 synovial thickness, wrist joint synovial thickness, MCP2 synovial blood flow integrity and carpal synovial blood flow scores all improved significantly following the treatment (Table I).

### Transversal correlation between ultrasonic variables and disease activity

Prior and subsequent to treatment, the DAS28 was strongly correlated to the MCP2 synovial thickness, wrist joint synovial thickness and MCP2 synovial blood flow integrity (Table II). Although wrist joint synovial blood flow integrity prior to treatment had no correlation with DAS28 (r=0.076, P=0.694), the index following the treatment showed a strong correlation (r=0.727, P<0.0001).

MCP2 synovial blood flow integrity also showed a correlation with CRP levels (r=0.372, P=0.047 prior to treatment; r=0.382, P=0.041 subsequent to treatment), but each ultrasonic parameter had no statistically significant correlation with the ESR (Table III).

## Discussion

Accurate monitoring of disease progression is the key to successful RA treatment. Therefore, rheumatologists must closely monitor fluctuations in RA parameters and tailor treatment to account for changes in patient disease progression. In the past few years, RA inflammation has been indirectly evaluated using clinical examination (such as DAS28) and laboratory tests. The development of novel iconographical technologies, particularly muscular and skeleton ultrasound, has allowed clinicians to perform more accurate follow-up evaluations of RA patients. Since ultrasound is relatively simple, direct and cheap, and has no risk of radiation, it is more likely to be accepted by the patient. Furthermore, ultrasound examination can be used repeatedly so it is more suitable for follow-up monitoring than other methods, such as magnetic resonance imaging (MRI). Simultaneously, previous studies have shown that ultrasound and MRI is highly consistent in their ability to diagnose early RA (7–9). The present investigators are currently conducting studies on imaging in the diagnosis of RA, and have found that ultrasonography diagnosis of synovitis and early bone damage is more accurate than X-ray, and it is more cost effective than MRI (it costs ¥200 compared to ¥900). Furthermore, ultrasonography has been increasingly accepted by clinicians, and has become one of the standard methods of evaluating RA by imaging (10–13).

There are a number of different ultrasonic testing methods for therapeutic evaluation of RA worldwide, including two-dimensional gray scale ultrasound, color Doppler flow imaging, color Doppler energy, spectral Doppler ultrasound, contrast-enhanced ultrasound and 3D ultrasonic methods. Some of these methods use the wrist, the 2 and 3 metacarpophalangeal joints and the proximal interphalangeal joint as testing objects, whereas others use the knee, ankle, elbow and metatarsophalangeal joints (4,14–18). In addition, the most common pathological changes observed in the ultrasonic detection of RA disease joints are synovitis and bone erosion. Other inflammatory changes also include bursitis, tendonitis and arthroedema. Rheumatologists in different countries and regions have created their own quantitative evaluation criteria, and these criteria are not uniform. Therefore, it is necessary to formulate simple, feasible and accurate ultrasonic testing methods for their ability to evaluate RA in clinical practice.

In the present study, the synovitis was selected as the main index, and the synovial thickness and synovial color Doppler scoring was measured. The reason for not including bone erosion as an evaluation index was due to the ultrasound grading system of bone erosion having a strong acoustic window dependency, the restriction of the checkpoint and the bone erosion evaluation was not as accurate as MRI. When the sensitivity of gadolinium-enhanced nuclear magnetic resonance was compared to power Doppler ultrasound in its ability to analyze synovitis, the power Doppler ultrasound was 88.8% as effective as the MRI, and the specificity of the Doppler was 97.9% (19). The pannus formation was the pathological basis of RA synovitis. In the pathogenesis of RA, synovial angiogenesis appears extremely early, and has already existed even prior to the inflammation results in clinical symptoms and histological changes (20–22). The appearance of Doppler-detected synovitis and histopathologically-confirmed synovial changes was high (23). There is no uniform standard in the color Doppler scoring of synovitis, but the Stone standard, the Szkudlarek Standard established in 2001 and the Larche Standard established in 2010 are commonly used. In the present study, the Szkudlarek standard was used, which exhibited good maneuverability.

There remains a huge dispute regarding the number of joints that should be included in the evaluation and which regions should be scanned. Scheel *et al* (24) established an ultrasound scoring system of RA finger arthrosynovitis, which recommended that the joints of MCP2–4 and PIP 2–4 (proximal interphalangeal joint) are examined. In another study by Szkudlarek *et al* (22), ultrasound was shown to have repeatability and observer consistency on the finger and toe joint synovitis using a semi-quantitative method. Furthermore, Naredo *et al* (25) performed an exploratory analysis of 128 sites of 44 joints, and found that the ultrasound examination and synovial scoring of the 24 sites of 12 joints (bilateral elbow, wrist, MCP2–3, knee and ankle joints) were also simple and effective. The wrist, MCP2 and proximal interphalangeal joints are widely accepted as the earliest and most commonly affected joints. Thus, in order to reduce the examination time, only the unilateral wrist and MCP2 joint were included into the present study. This allowed repeated examinations on only a few joints, with the assumption that the selected joints had the synovitis.

Therefore, in the ultrasound evaluation system described in the present study, the high frequency color Doppler ultrasound was chosen as it had wide application, reliable quality and convenient operation. A single MCP2 and the wrist as target structure were used, focusing on the detection of synovial thickness and synovial blood flow.

The results revealed that 29 of the 36 patients recruited completed the study, and all the research indicators showed a significant difference prior and subsequent to the treatment. The study showed that the 29 subjects showed improvement in their disease after 24 weeks of treatment, and that the unilateral MCP2 and carpal synovial thickness, synovial blood flow integral and MCP2 correlated with DAS28. The synovial color Doppler blood flow scoring of MCP2 and wrist exhibited an improved correlation with DAS28 than the laboratory indexes, including blood sedimentation and CRP. Naredo *et al* (26) performed a 1-year follow-up on early-stage RA patients and found that compared with clinical performance and laboratory indexes (blood sedimentation and CRP), the ultrasonic synovial joint number and power Doppler ultrasound synovial scoring exhibited a strong correlation with the DAS scoring and iconographical progress. Naredo *et al* (27) also performed a 10-joint ultrasound examination of JIA patients, and the study revealed that ultrasound is convenient and feasible. Although the study only performed the evaluation of two ultrasonic indexes of two joints, the ultrasound data were consistent with the DAS28 data, and the results were consistent with previous studies (11,28,29).

In the present study, approximately two-thirds of the patients were treated with a combination of TNFα receptor antagonist and amethopterin. There are several cohort studies that have shown that treatment of RA patients with TNFα antagonist results in significant improvement in disease when measured by ultrasound (27,30–32). An evaluation of TNFα receptor antagonist efficacy in the treatment of RA patients revealed that the synovial color Doppler scoring of 28 joints was parallel to the changes in DAS28. Therefore, the ultrasonic inflammatory joints number and synovial color Doppler scoring was predictive of X-ray bone erosion (32). Scirè *et al* (33) performed a 2-year follow-up of 106 cases of early-stage RA patients, and showed that the exploration of color Doppler ultrasound towards the joint residual inflammation was much more sensitive than the clinical and laboratory indexes. The present study also found that the ultrasound detected the residue of focal arthrosynovitis in patients who showed disease remission as determined by DAS28. The study also revealed the parallel between the synovial color Doppler scoring and the changes in DAS28. However, since there were a fewer number of joints examined in the present study, the bone erosion examination was disregarded and the observation time was only 24 weeks. Thus, the contrasting observations prior and subsequent to the X-ray experiment could not be carried out. The association between synovial color Doppler scoring and bone erosion can be observed and is the focus of our future research.

In the present study, carpal synovial blood flow integrity also showed a good correlation with DAS28 prior and subsequent to treatment, however, the results prior to treatment may be quite different since the sample size was small and there may be deviation of individual study data. The small sample size is one of the limitations of the study, and further studies are required.

ESR and CRP were noted to be extremely important markers of inflammation, and they showed a strong correlation with DAS28 in the study. Although ESR and CRP can be used as biomarkers of RA severity, the ultrasonic index failed to exhibit correlation with ESR or CRP, indicating that DAS28 is a more reliable correlate than ultrasound index. Again, the small size of the sample population in the study may influence this observation.

In conclusion, the results of the present study confirmed that ultrasound examination and evaluation of a few joints was feasible and predictive, and was consistent with the RA disease state as measured by DAS28. The use of this ultrasound method is quick, convenient and clinically applicable. Since RA is heterogeneous and involves numerous different joints, the number of joints evaluated in the present study was lower. This may reduce the specificity of the analysis, and may introduce a certain degree of error. The study did not examine bone erosion, thus the prevention of disease recurrence and prediction of bone erosion were limited. In future studies, larger sample size, evaluation of a greater number of joints (such as the interphalangeal joint) and measuring bone erosion in order to determine the optimum method for ultrasonic inspection of RA are required to make it more feasible and reliable in clinical practice.

## Figures and Tables

**Table I:** Comparison of the index of DAS28, ESR, CRP, MCP2 synovial thickness, wrist joint synovial thickness and MCP2 synovial blood flow integral before and after 24 weeks treatment with the carpal synovial blood flow integral.

	Statistic treatment description			
			
Index	Prior	Subsequent	Statistics	P-value
DAS28	6.10±0.90	3.56±1.29	−12.080	<0.0001
CRP, mg/l	16.20±25.77	3.34±0.93	−30442	0.001
ESR, mm/h	34.00±30.00	20.00±27.00	−4.045	<0.0001
MCP2 synovial thickness, mm	3.35±0.66	2.80±0.60	−4.506	<0.0001
Wrist synovial thickness, mm	4.21±0.69	3.30±1.00	8.957	<0.0001
MCP2 synovial blood flow integral	3.00±1.00	1.00±1.00	−4.626	<0.0001
Wrist synovial blood flow integral	2.00±1.00	1.00±1.00	−3.257	<0.0001

DAS28, 28-joint disease activity score; CRP, C-reactive protein; ESR, erythrocyte sedimentation rate; MCP2, metacarpophalangeal second joint 2.

**Table II:** Correlation between MP2 synovial thickness, wrist synovial thickness, MP2 synovial blood flow integral and wrist joint synovial blood flow integral of prior and subsequent to treatment and DAS28.

	Prior to treatment		Subsequent to treatment	
		
Index and DAS28	γ	P-value	γ	P-value
MP2 synovial thickness	0.804	<0.0001	0.591	0.001
Wrist synovial thickness	0.534	0.003	0.403	0.030
MCP2 synovial blood flow integral	0.456	0.013	0.807	<0.0001
Wrist synovial blood flow integral	0.076	0.694	0.727	<0.0001

MCP2, metacarpophalangeal second joint 2; DAS28, 28-joint disease activity score.

**Table III:** Correlation between MCP2 synovial thickness, wrist joint synovial thickness, MCP2 synovial blood flow integral and wrist joint synovial blood flow integral for prior to and following treatment and ESR and CRP.

	Prior to treatment		24 weeks after treatment	
		
Index and ESR or CRP	γ	P-value	γ	P-value
ESR				
MCP2 synovial thickness	0.485	0.008	0.350	0.063
Wrist synovial thickness	0.459	0.012	0.156	0.419
MCP2 synovial blood flow integral	0.229	0.231	0.355	0.059
Wrist synovial blood flow integral	−0.014	0.941	0.564	0.001
CRP				
MCP2 synovial thickness	0.397	0.033	0.248	0.195
Wrist synovial thickness	0.083	0.669	0.142	0.463
MCP2 synovial blood flow integral	0.372	0.047	0.382	0.041
Wrist synovial blood flow integral	0.195	0.310	0.355	0.058

MCP2, metacarpophalangeal second joint 2; ESR, erythrocyte sedimentation rate; CRP, C-reactive protein.
